# Multidisciplinary consensus on the criteria for fertility preservation in cancer patients

**DOI:** 10.1007/s12094-021-02699-2

**Published:** 2021-10-11

**Authors:** A. Santaballa, C. Márquez-Vega, Á. Rodríguez-Lescure, Á. Rovirosa, L. Vázquez, I. Zeberio-Etxetxipia, M. Andrés, L. Bassas, E. Ceballos-Garcia, J. Domingo, D. Manau-Trullas

**Affiliations:** 1 Servicio de Oncología Médica, Sociedad Española de Oncología Médica (SEOM), Hospital Universitario y Politécnico de La Fe, Avenida Fernando Abril Martorell, 106, 46026 Valencia, Spain; 2 Sociedad Española de Hematología y Oncología Pediátricas (SEHOP), Hospital Universitario Virgen del Rocío, Seville, Spain; 3 Sociedad Española de Oncología Médica (SEOM), Hospital General Universitario de Elche, Elche, Spain; 4 Sociedad Española de Oncología Radioterápica (SEOR), Hospital Clinic, Barcelona, Spain; 5 Sociedad Española de Hematología y Hemoterapia (SEHH), Hospital Universitario de Salamanca, Salamanca, Spain; 6 Sociedad Española de Hematología y Hemoterapia (SEHH), Hospital Universitario Donostia, San Sebastián, Spain; 7 Sociedad Española de Hematología y Oncología Pediátricas (SEHOP), Hospital Universitario y Politécnico de La Fe, Valencia, Spain; 8 Sociedad Española de Fertilidad (SEF), Servicio de Andrología, Laboratorio de Andrología y Banco de Semen, Fundación Puigvert, Barcelona, Spain; 9 Sociedad Española de Fertilidad (SEF), Hospital Universitario Gregorio Marañón, Madrid, Spain; 10 Sociedad Española de Fertilidad (SEF), IVI Las Palmas, Las Palmas de Gran Canaria, Spain; 11 Sociedad Española de Fertilidad (SEF), Unidad de Reproducción Humana Asistida, Hospital Clinic, Barcelona, Spain

**Keywords:** Cryopreservation, Male and female infertility, Gonadotoxicity, Live birth, Pregnancy, Cancer patients

## Abstract

Infertility is one of the main sequelae of cancer and its treatment in both children and adults of reproductive age. It is, therefore, essential that oncologists and haematologists provide adequate information about the risk of infertility and the possibilities for its preservation before starting treatment. Although many international clinical guidelines address this issue, this document is the first Spanish multidisciplinary guideline in paediatric and adult oncological patients. Experts from the Spanish Society of Medical Oncology, the Spanish Fertility Society, the Spanish Society of Haematology and Haemotherapy, the Spanish Society of Paediatric Haematology and Oncology and the Spanish Society of Radiation Oncology have collaborated to develop a multidisciplinary consensus.

## Introduction

The survival of patients with cancer has increased in recent years and is expected to continue improving [1, 2]. Five percent of cancer survivors are under the age of 40 years. The assessment of long-term side effects in this population is important to provide the best quality of life for these survivors. Infertility is one of the main sequelae of cancer, and its treatment in both children and adults of reproductive age has a great impact on quality of life. Oncologists and haematologists must adequately inform patients about the potential risk of infertility and the current possibilities for preserving fertility before starting treatment. The prospect of preserving fertility has beneficial psychological effects for patients, helping to boost their confidence in the treatment and project positive personal goals into the future [3].

While many international clinical guidelines address fertility preservation in cancer patients, this document is the first Spanish multidisciplinary guideline in paediatric and adult oncological patients. For this reason, the Spanish Society of Medical Oncology (SEOM), the Spanish Fertility Society (SEF), the Spanish Society of Haematology and Haemotherapy (SEHH), the Spanish Society of Paediatric Haematology and Oncology (SEHOP) and the Spanish Society of Radiation Oncology (SEOR) have collaborated to review the current evidence on this issue in paediatric, adolescent, and adult patients with cancer, and to develop a multidisciplinary consensus.

## Gametogenesis

### Ovarian reserve

At birth, the ovaries have a limited number of oocytes that constitute the ovarian reserve. The follicular pool has around 7 million follicles in the 20th week of gestation, and this number decreases progressively until menopause. Only approximately 300 follicles will reach maturity during reproductive life.

Ovarian ageing results in a loss of fertility, reducing the likelihood of both spontaneous conception and pregnancy through assisted reproduction techniques. This is mainly due to the progressive decrease of the initial follicular pool (quantitative decrease), but also to the loss of oocyte quality, which increases the percentage of meiosis alterations (qualitative decrease) and results in aneuploid embryos. The concept of ovarian reserve, therefore, encompasses both the quantity and quality of a woman’s oocytes.

Currently, it is difficult to predict a woman's reproductive capacity. Several markers are used to estimate ovarian reserve, such as antral follicle count (AFC) and anti-Müllerian hormone (AMH) levels; however, their value for predicting pregnancy remains uncertain. Nonetheless, these markers are useful for predicting ovarian responsiveness to stimulation in an in vitro fertilisation cycle*.* Although the normal range of AMH in the general population is wide, in patients under 20 years of age the predictive value is lower; consequently, there is not a well-defined normal value for these patients. Therefore, when considering the indications for and performance of fertility preservation, both the age of the patient and her ovarian reserve must be assessed.

AMH levels before gonadotoxic treatment may correlate with subsequent ovarian function; however, their relationship with reproductive capacity after treatment is controversial. Fertility assessments must, therefore, be based on markers (AMH and AFC) together with age.

### Spermatogenesis

Spermatogenesis occurs in the seminiferous tubules of the testes and is the process by which the male primordial germ cells, spermatogonia, produce spermatozoa. It begins in puberty and persists throughout adult life. Spermatogonia differentiate to initiate meiosis, producing haploid spermatids and then spermatozoa, after complete differentiation. The maturation of sperm is completed in the epididymis. The entire process is controlled by Sertoli cells, which surround germ cells and promote their progression in response to testosterone, follicle-stimulating hormone (FSH), and multiple regulatory proteins.

Spermatogonia are susceptible to apoptosis induced by ionising radiation and cytostatics [4]. Spermatids are more resistant but have no DNA repair mechanisms. Tubular lesions are reflected by elevated serum FSH and are accompanied by a decrease in testicular volume and consistency due to germ cell depletion. Chemotherapy can also damage Leydig cells and temporarily or permanently decrease steroidogenesis, leading to elevated luteinising hormone (LH) levels [5]. Testicular volume, semen analysis and gonadotropin levels are the most useful tools for assessing the degree of testicular injury associated with gonadotoxic treatment.

## Gonadotoxicity of current treatments

**Fig. 1:**
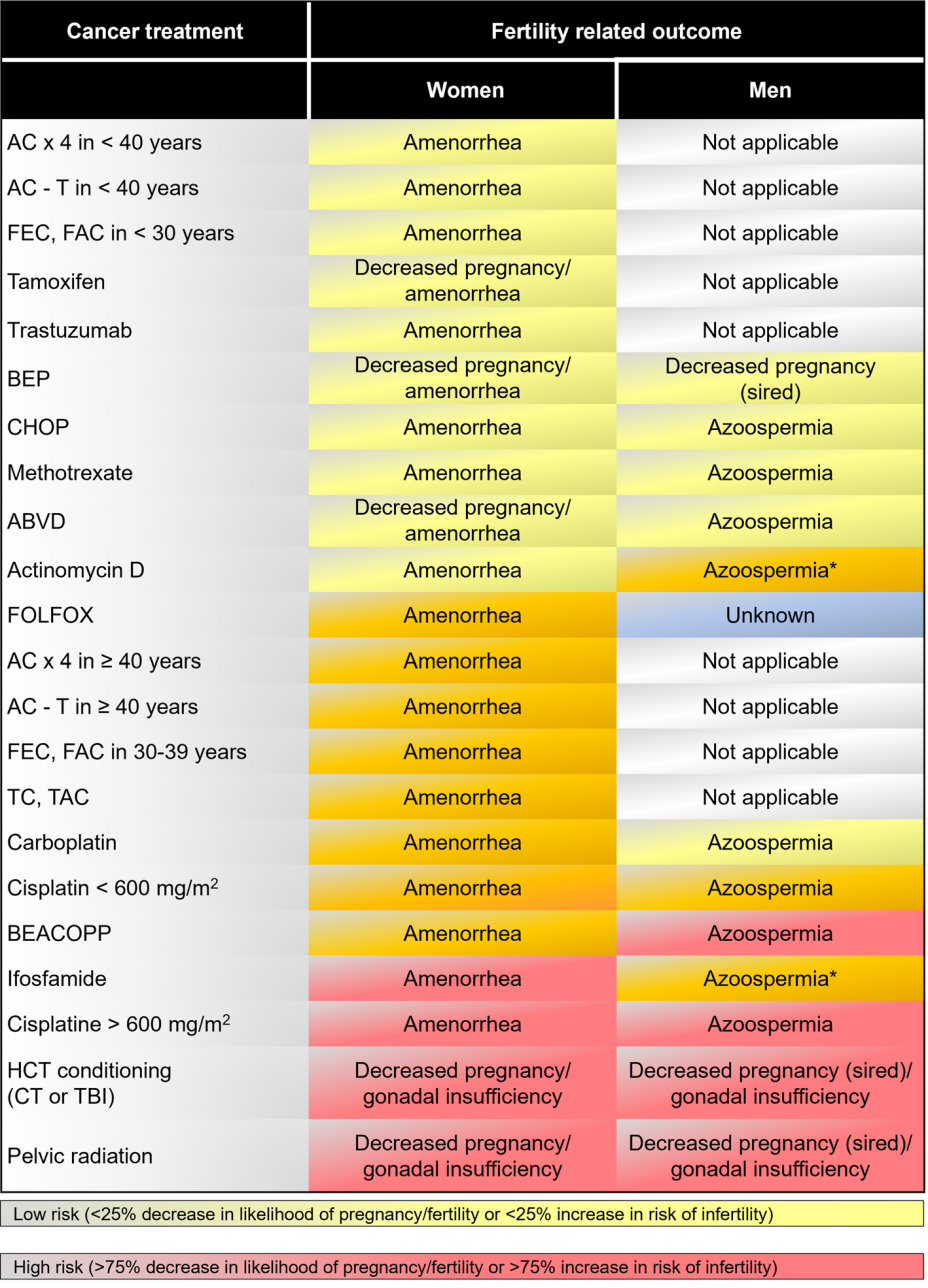
Risk of infertility associated with antineoplastic treatment in adult cancer patients. *Azoospermia likely when given with other highly sterilizing agents. *ABVD* doxorubicin (Adriamycin^®^), bleomycin, vinblastine and dacarbazine; *AC* doxorubicin (Adriamycin^®^) and cyclophosphamide; *AC-T* doxorubicin (Adriamycin^®^), cyclophosphamide and paclitaxel (Taxol^®^); *BEACOPP* bleomycin, etoposide, doxorubicin (Adriamycin^®^), cyclophosphamide, vincristine (Oncovin^®^), procarbazine and prednisone; *BEP* bleomycin, etoposide and platinum; *CHOP* cyclophosphamide, doxorubicin (Adriamycin^®^), vincristine and prednisone; *CT* chemotherapy; *FAC* fluorouracil, doxorubicin (Adriamycin^®^) and cyclophosphamide; *FEC* fluorouracil, epirubicin and cyclophosphamide; *FOLFOX* fluorouracil, leucovorin and oxaliplatin; *HCT* haematopoietic cell transplantation; *TAC* docetaxel (Taxotere^®^), doxorubicin (Adriamycin^®^) and cyclophosphamide; *TBI* total body irradiation; *TC* docetaxel (Taxotere^®^) and cyclophosphamide

**Fig. 2:**
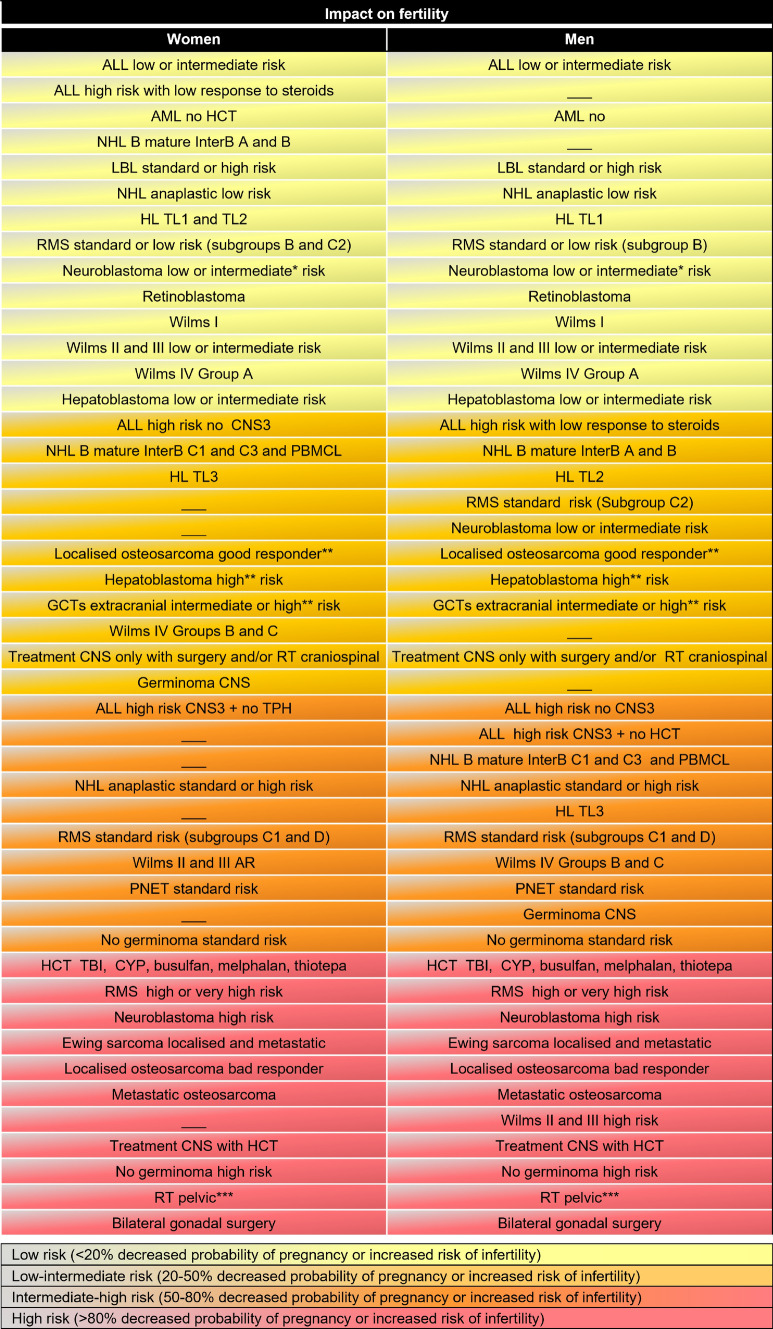
Risk of infertility associated with antineoplastic treatment in paediatric and adolescent patients. *ALL* acute lymphoblastic leukaemia; *AML* acute myeloid leukaemia; *CNS* central nervous system; *CNS3* CNS with cerebrospinal fluid 5 ≥ leukocytes/µl and blast positives *Colli et al., 205*; *CYP* cyclophosphamide; *GCTs* germ cell tumors; *HL* Hodgkin lymphoma; *HCT* hematopoietic cell transplantation; *HSCT* hematopoietic stem cell transplantation; *LBL* lymphoblastic lymphoma; *NHL* non-Hodgkin lymphoma; *PMBCL* primary mediastinal B-cell lymphoma; *PNET* primitive neuroectodermal tumour; *RMS* rhabdomyosarcoma; *RT* radiotherapy; *TBI* total body irradiation. To calculate the gonadotoxic risk [70–72], the patient should receive an equivalent dose of CFM (cyclophosphamide equivalent dose, CED) according to the formula of Green et al.: > 6000–8000 mg/m^2^ in women and > 4000 mg/m^2^ in men, ovarian or testicular RT or HSCT (73). The following equivalences were used (CFM = 1; ifosfamide × 0.244; procarbazine × 0.857; chlorambucil × 14.28; BCNU × 16; melphalan × 40; thiotepa × 40; nitrogen mustard × 100; busulfan × 8.82). In women, considered: low risk (CED < 4000 mg/m^2^), low-intermediate risk (CED between 4000 and 6000 mg/m^2^), intermediate-high risk (CED between 6000 and 10,000 mg/m^2^), high risk (CED > 10,000 mg/m^2^). In men, considered: low risk (CED < 2000 mg/m^2^), low-intermediate risk (CED between 2000 and 4000 mg/m^2^), intermediate-high risk (CED between 4000 and 8000 mg/m^2^), high risk (CED > 8000 mg/m^2^). *Depending on the dose of alkylating agent used. **Risk calculated by cumulative dose of platinums (gonadotoxic potential not agreed). ***Pelvic RT (> 15 Gy in prepubertal and > 10 Gy in postpubertal) or total abdominal RT. Craniospinal RT if the ovaries (> 2 Gy) or testes are included in the field (0.1–1.2 Gy)

In the adult population, gonadal damage is difficult to predict accurately. This damage can be caused by surgery, radiotherapy, chemotherapy or all of these factors together. Overall gonadotoxicity figures after paediatric cancer range between 8 and 30%, although they can increase to 70–90% in high-risk subgroups. Figure 1 describes the risk of infertility associated with anticancer treatments in adults, and Fig. 2 describes the risk in children and adolescents.

### Surgery

The surgeries that are necessary to treat some tumours compromise or remove the reproductive organs, diminishing or eliminating the fertility of affected patients [6].

The need for bilateral gonadal excision as part of cancer treatment in the paediatric population is rare. However, on occasion, the hypothalamic-pituitary stalk can be affected by surgical treatment for some brain tumours, producing an alteration in gonadotropins and potentially affecting fertility.

### Radiotherapy

The impact of radiotherapy on fertility is variable, and sometimes it is impossible to establish a prognosis. Intensity-modulated radiotherapy should always be used to preserve reproductive tissues, and radiotherapy doses received by the reproductive organs must be assessed when planning a future pregnancy (Fig. 1).

Radiotherapy impacts the fertility of children and young people with acute leukaemia, lymphomas, Wilms tumours, pelvic sarcomas and brain and nasopharyngeal tumours. In adults, it affects patients with rectal, sigmoid and anal cancer, cervical and uterine cancer, sarcomas, leukaemia, lymphomas and brain and nasopharyngeal tumours.

In women, radiation can alter fertility by inducing damage at three levels: (i) the pituitary axis, which can be affected by both cranial and total body irradiation, causing infertility, miscarriage, delayed puberty and hypogonadotropic hypogonadism; replacement therapy and ovulation induction with gonadotropins will be required if there is a desire for pregnancy; (ii) the ovaries, where the damage depends on the dose of radiation and whether it is combined with chemotherapy and causes menstrual irregularities, acute ovarian failure, decreased ovarian reserve, infertility, miscarriage, early menopause and delayed puberty; damage to the ovaries results from pelvic, spinal, total abdominal and total body irradiation (similarly for uterine damage) and the dose that causes ovarian failure varies according to age, with adult patients being more sensitive to radiation than adolescents or children; the dose that produces a 50% decrease in ovarian reserve is ≤ 2 Gy; (iii) uterine damage, which is associated with infertility, miscarriage, preterm delivery, low birth weight, abnormal placental implantation, foetal malposition and intrauterine and perinatal death, as well as increased rates of diabetes, hypertension and eclampsia during pregnancy; depending on the administered doses, different degrees of blood supply alteration, decreased uterine size and elasticity, myometrial fibrosis and necrosis and endometrial atrophy with the replacement of tissue by dense collagen depositions can occur; the cervix becomes atrophic and loses elasticity (mainly in adults); additionally, implantation is hindered, and although the dose that prevents pregnancy is unknown, pregnancy has been considered unviable at doses > 25 Gy.

In men, radiotherapy affects the testicular germinal epithelium, including Sertoli cells and, to a lesser extent, Leydig cells, which may impair spermatogenesis; it does not usually cause hypogonadism, but the effects on spermatogenesis depend on the dose and the regimen used (doses ≥ 1.2 Gy are associated with azoospermia, which is permanent at doses > 6 Gy) [7].

### Hormone therapy

Although hormone therapy is not gonadotoxic per se, increased treatment duration may increase the risk of infertility due to the decrease in ovarian reserve with age. Patients should be informed of this risk when starting endocrine therapy, and the duration of the treatment should be considered [8–13].

Ovarian suppression with luteinising hormone-releasing hormone (LHRH) analogues combined with tamoxifen or aromatase inhibitors is an adjuvant treatment for patients with hormone receptor-positive breast cancer. Published data estimate that ovarian function usually recovers approximately 3 months after treatment is completed [14].

Androgen deprivation therapy causes hypogonadism and low testosterone levels; consequently, it can be associated with oligospermia and azoospermia and cause transient sterility.

### Chemotherapy

Chemotherapy, especially with alkylating agents, produces gonadotoxicity in both sexes. The risk of gonadal toxicity depends on the type of treatment received (Fig. 1), the accumulated dose, the state of the gonads before treatment begins and, in particular, the patient’s age when chemotherapy is administered [15].

Chemotherapy affects the germinal epithelium more than the Leydig cells; consequently, men receiving chemotherapy may have oligospermia, azoospermia or partial or compensated hypogonadism.

Chemotherapy can affect primordial follicles but may also reduce the number of growing follicles, where a “burn-out” effect may occur because of accelerated follicular recruitment which can lead to premature ovarian failure. Since the ovarian reserve decreases with age, the risk of permanent ovarian failure increases in older women. Additionally, vascular damage, stromal injury or fibrosis may occur.

### Immunotherapy and other targeted therapies

Available data on the infertility risk associated with biological treatments are scarce and heterogeneous [16]. Imatinib does not cause infertility in men or women. Data on nilotinib and dasatinib suggest that they do not affect gonadal function. However, data from the Summary of product characteristics (SmPC) and clinical trials confirm that the use of tyrosine kinase inhibitor*s* (TKI) is contraindicated during pregnancy. Although angiogenesis plays a crucial role in gonadal development, preclinical studies show that male and female fertility is only moderately affected by sunitinib and other TKIs with antiangiogenic activity, such as sorafenib or pazopanib.

There are no preclinical studies on the effect of bevacizumab on fertility. A clinical trial of the addition of bevacizumab to adjuvant chemotherapy in colon cancer found that the incidence of ovarian failure in premenopausal women was 3% when bevacizumab was not administered and 39% when it was. However, at the end of treatment with bevacizumab, 86% of patients regained ovarian function [17].

Trastuzumab and lapatinib do not confer an increased risk of infertility after chemotherapy [18]. In preclinical models with other EGFR TKIs, such as erlotinib or gefitinib, a decrease in fertility parameters was observed, although the effect in humans is unknown. The only clinical study with gefitinib shows suppression of androgen levels in both men and women.

Clinical studies evaluating the effect of everolimus on gonadal function have been conducted in men who have undergone kidney transplant, but not in cancer patients. These studies show that everolimus produces a decrease in testosterone levels and disrupts spermatogenesis.

According to preclinical data, crizotinib can cause infertility in men and women. Clinical data indicate crizotinib lowers testosterone levels, which should be monitored in males receiving this treatment.

There is very little evidence regarding the possible gonadal toxicity of immunomodulatory drugs, including immune checkpoint inhibitors such as ipilimumab, nivolumab, pembrolizumab, atezolizumab, avelumab and durvalumab. Therefore, the risk of gonadotoxicity remains unknown [19]. Some patients will develop immunity-related adverse events, such as hypothyroidism or hypophysitis, which can cause ovarian failure and decreased testosterone levels.

## Fertility preservation techniques

Currently, there are several techniques for preserving fertility, such as conservative surgery of the reproductive organs in the early stages of disease and cryopreservation techniques (of embryos, oocytes, ovarian cortex, semen and testicular tissue). The paediatric population is particularly vulnerable, and practical, ethical and legal factors must be considered in addition to strictly biological factors before applying these procedures [20, 21].

Table 1 describes the main indications for fertility preservation in paediatric, adolescent and adult patients with cancer.

**Table 1:** Indications for offering fertility preservation in paediatric, adolescent and adult patients with cancer or haematological disease

Females: > 2 years and < 35–40 years with preserved ovarian reserve^a^
Males: advanced or complete puberty, presence of spermatozoa in semen or testicles
Patients diagnosed with cancer or haematological disease whose curative treatment has an intermediate-high gonadotoxic risk (> 50–80%)
Patients diagnosed with cancer or haematological disease whose curative treatment has a low-intermediate gonadotoxic risk (20–50%) but who have certain additional conditions (i.e., low ovarian reserve, previous ovarian surgery, cryptorchidism, monorchidism, previous testicular injury, age, etc.)
Patients who have not received previous gonadotoxic chemotherapy or have received only low doses^b^
Patients receiving first-line treatment with curative intent

^a^In patients under 2 years of age, the indication must be individualised. In women over 35 years of age, the ovarian reserve should be assessed

^b^Provided the ovarian reserve is preserved (females) or the risk of genetic damage is low (males)

### Surgical techniques

Ovarian transposition or oophoropexy is a surgical procedure indicated when pelvic radiotherapy is administered without chemotherapy or with a low-gonadotoxic one. This technique has been used in cases of cervical and rectal cancer, lymphomas and other tumours. Transposition above the iliac crests is recommended to move the ovary away from the radiation field; this procedure can preserve ovarian function in 50–80% of cases.

Ovarian transposition can be combined with other techniques, such as ovarian cortex or oocyte preservation, since its effectiveness may be limited and can hinder subsequent assisted reproduction techniques [22, 23].

### Oocyte cryopreservation

Oocyte cryopreservation is currently the fertility preservation technique of choice [24]. The ideal number of mature oocytes that should be retrieve for vitrification is a controversial issue. Cryopreservation of between 10 and 15 oocytes is recommended as the probability of pregnancy is higher with a greater number of oocytes; however, this correlation is not so clearly observed after the age of 35 years due to the effect of age on oocyte quality and the increase in aneuploidies.

Ovarian hormonal stimulation using gonadotropins is required and usually lasts between 10 and 12 days, at the end of which a transvaginal ovarian puncture with follicular aspiration is performed under sedation for oocyte retrieval. Subsequently, the patient can start chemotherapy without delay. Random-start stimulation may be initiated at any time during the patient’s ovarian cycle [25]. In cases of low response, if the start of chemotherapy allows, several stimulation or double stimulation cycles can be performed in the same cycle (DuoStim) to increase oocyte availability [26]. The complications of this procedure are the same as those associated with oocyte retrieval: hemoperitoneum, ovarian torsion, infection or hyperstimulation syndrome (minimal with the use of GnRH agonists to achieve ovulatory discharge), although these are rare (< 1%) in the absence of risk factors (e.g., coagulation alterations, etc.).

In adolescent patients, oocyte cryopreservation is physiologically feasible starting at puberty, considering the particularities of this population.

### Ovarian tissue cryopreservation

This experimental technique is indicated for patients who must urgently start chemotherapy, prepubertal patients and patients with hormone-dependent cancers with contraindications for hormonal stimulation. It is more effective in patients under 35 years of age [27].

Ovarian tissue cryopreservation requires a double surgical procedure. The extraction (partial oophorectomy, total oophorectomy, decortication) and reimplantation (in the contralateral ovary or pelvic wall) techniques are not standardised. Histopathological analysis should be routinely performed to rule out the possibility of micrometastases. The loss of functional capacity of the tissue occurs mainly after reimplantation due to ischaemia and subsequent follicular burn-out. More than 100 live births conceived spontaneously or through assisted reproduction have been reported as a result of this technique.

Ideally, the patient should not have undergone any previous chemotherapy regimen; however, the possibility of ovarian cryopreservation can be assessed even if the gonadotoxic risk is very high [20, 28]. Among the main limitations of this technique are the theoretical risk of reinserting tumour cells; therefore, it is contraindicated in patients with leukaemia or central nervous system tumours and is still considered an experimental technique in some countries. In prepubertal patients, it is the only fertility preservation option available. It could potentially be used from birth, but there are few data in children under 5 years of age, although some groups offer it for patients older than 1 year [28]. Because it is experimental, it is important to perform it with an alternative anaesthetic procedure.

### Semen cryopreservation

Semen freezing should be performed as soon as possible after ejaculation once liquefaction and semen evaluation have been completed. Rapid freezing (−50 °C per minute) is performed manually by depositing the containers in the nitrogen vapour phase for 30 min and then quickly immersing the straws/vials in liquid nitrogen.

Sperm vitrification is an alternative cryopreservation method for which limited reports of clinical experiences are available. It is indicated for low-volume samples with a low sperm count and poor sperm quality, as it seems to preserve vitality and DNA integrity better than classical cryopreservation [29]. Sperm cryosurvival is assessed by measuring the degree of sperm motility in an aliquot after thawing and comparing the results to fresh-sample motility.

For patients with anejaculation, available alternatives include penile vibratory stimulation, transrectal electroejaculation under general anaesthesia, epididymal aspiration and testicular biopsy. The latter technique is also indicated for azoospermic patients and those without any sperm mobility in the cryosurvival test.

Semen cryopreservation should be offered before starting any therapy. Patients who are referred to a semen bank after they have started treatment may find that freezing is no longer an option due to the absence of sperm in the ejaculate or very poor semen quality. Acute effects of genotoxic treatments include genetic mutation, sperm DNA fragmentation, chromosome breakage and sperm aneuploidy. With alkylating agents, the risk of genetic damage is greatest at day one of treatment and persists for at least one spermatogenic cycle (75 days). In the case of radiotherapy, the maximum risk occurs one week after the start of the doses, but it persists for up to 2 months. Topoisomerase II inhibitors act mainly on meiosis; consequently, the greatest damage occurs between 30 and 50 days after administration [30]. These circumstances, as well as the inevitable effect of the underlying disease (hormonal or paracrine dysregulation in testicular tumours, fever in lymphomas or leukaemia, radiation exposure from diagnostic tests) are individual risk factors that should be included in the consent form.

In young people who have entered puberty and are at Tanner stage > 2 and have a testicular volume > 8–10 mL, it is possible to find mature sperm in semen. However, these patients can be very sensitive to the pressure of their environment (e.g., from family or professionals) during this period of the beginning of their sexual activity. In patients with unclear pubertal development (10–12 years), it is advisable to perform a hormonal assessment and a physical examination or ultrasound to estimate testicular volume. Examination of nocturnal urine for sperm (spermaturia) can be used as a non-invasive indicator of sexual maturity. If ejaculate collection is not possible and the examination suggests that pubertal development is advanced, a testicular biopsy may be performed.

### GnRH analogues

The use of GnRH analogues is an experimental technique, and their possible protective mechanism is unknown. Various mechanisms have been proposed, such as the inhibition of FSH secretion, a decrease in utero-ovarian blood flow [31] and the activation of GnRH receptors. There are numerous studies with conflicting results, most of which do not have good study design or adequate sample size. The main advantage of this technique is the simplicity of administering it along with cancer treatment, without a need to postpone treatment.

The main medical societies—the American Society of Clinical Oncology (ASCO), European Society of Medical Oncology (ESMO), European Society of Human Reproduction and Embryology (ESHRE) and SEOM—do not recommend the use of GnRH analogues as the only fertility preservation technique [6, 32–35] given the low level of evidence regarding their effectiveness. This technique may be an option for patients with hormone receptor-negative breast cancer for whom other techniques are not considered or for the preservation of ovarian function.

### Other experimental techniques

#### Techniques for female patients

##### In vitro oocyte maturation

In vitro maturation (IVM) is an alternative method that can help to avoid delays in the administration of oncological treatment. It is a viable option for patients with cancer since it consists of the puncture-aspiration of an unstimulated ovary and subsequent in vitro oocyte maturation. There are few data on children born after the use of this technique. IVM can cause several undesirable effects at the oocyte level and should, therefore, be considered an experimental technique [35].

##### In vitro development of primordial follicles

A dynamic multi-step culture system is necessary to support each of the follicle transition stages. This in vitro follicular growth approach must fulfil the changing requirements of the developing oocyte and its surrounding granulosa cells to maintain the interactions among these cells. This technique presents many challenges, such as the determination of the competence of the developing oocyte and the associated genomic imprinting [36].

#### Techniques for male patients

Fertility preservation in children and adolescents who have not entered puberty is still in the research and development stage and is far from achieving effective results in humans [37]. The most promising approach involves the autologous transplantation of spermatogonia of the immature testicular tissue, with the aim of colonising the seminiferous tubules by reimplantation through the rete testis and thus partially restoring spermatogenesis in vivo. The culture must be free of infiltration by malignant cells [38].

An alternative is the grafting of testicular tissue fragments. In this way, and under the appropriate environmental conditions, the architecture and paracrine interactions of the seminiferous tubules can be maintained. The third strategy is in vitro spermatogenesis from spermatogonia or even from embryonic stem cells or somatic cells undergoing cell reprogramming using artificial co-culture systems that mimic the structure of the original testis [39].

## Indications for fertility preservation

**Fig. 3:**
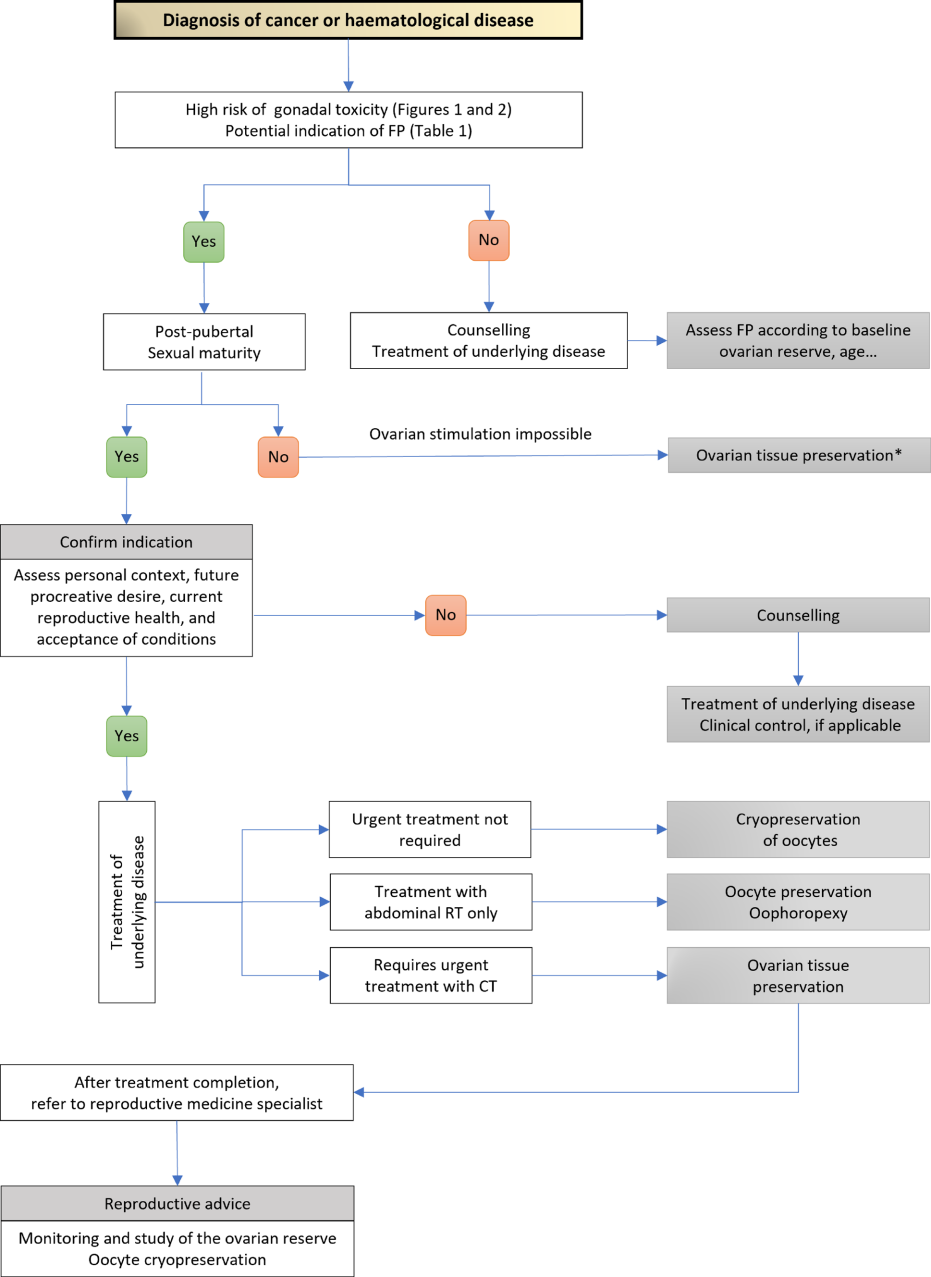
Decision-making algorithm to preserve fertility in female patients with cancer or haematological disease. *FP* fertility preservation; *CT* chemotherapy; *RT* radiotherapy. *Re-grafting is contraindicated in diseases with a high risk of ovarian metastasis, such as leukaemia, neuroblastomas or Burkitt’s lymphoma

**Fig. 4:**
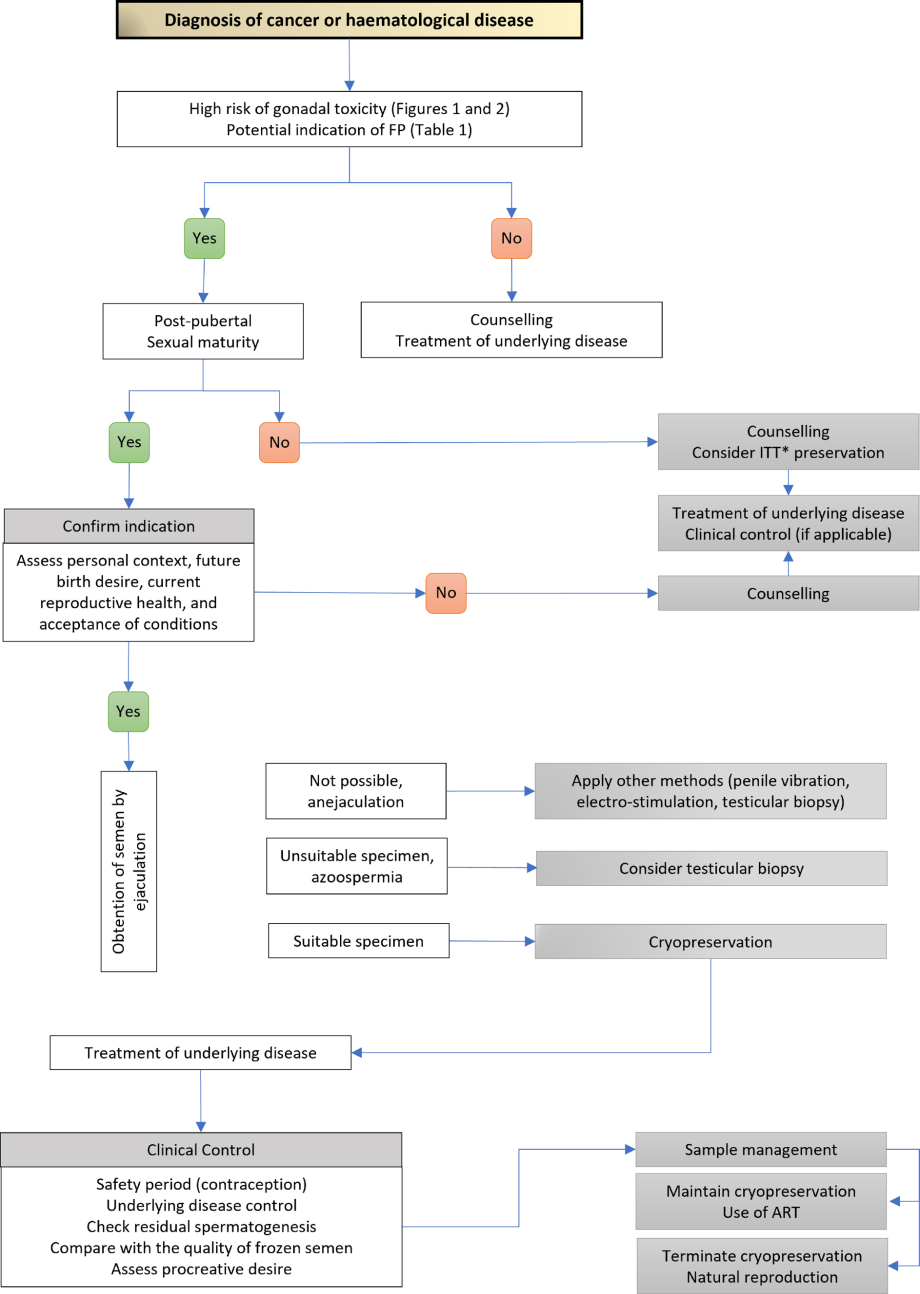
Decision-making algorithm to preserve fertility in male patients with cancer or haematological disease. *FP* fertility preservation; *ART* assisted reproductive techniques; *ITT* immature testicular tissue. *Considered experimental

Figures 3 and 4 show the decision-making algorithms to preserve fertility in female and male patients with cancer or a haematological disease.

### Paediatric and adolescent patients with cancer (<16 years)

After the initial diagnosis, patients and their families should receive an individualised assessment of the gonadotoxic risk of the treatment to be given and information about available fertility preservation options. During this process, both intrinsic factors (tumour type, location and stage, and patient age, gonadal function, pubertal stage before treatment and overall health status) and extrinsic factors (type of treatment, dose, time available, availability of qualified centres, parental consent, etc.) should be considered.

Table 1 outlines the main indications for fertility preservation in this population subgroup, in which the main and most complex factor is the calculation of the gonadotoxic risk associated with treatment (Fig. 2). Usually, this calculation takes into account the classification of Wallace et al. (2005) based on treatment intensity, which classifies infertility risk groups as low (< 20%), medium (21–80%) or high (> 80%) [21]. Over time, this classification has been modified according to long-term follow-up data, resulting in changes to some international strategies that encourage the selection of less gonadotoxic treatments, such as a procarbazine substitution, or avoiding radiotherapy if an adequate and rapid initial response is observed [20]. SEHOP, through the experience gained in the last 10 years with specific fertility preservation programmes in paediatric populations [40], has reclassified the risk subgroups by adding an intermediate category, resulting in the following classification: low < 20%, low-intermediate 20–50%, intermediate-high 50–75%, and high > 75%. This reclassification is based on the protocols used in Spain and was undertaken to provide a consensus and to standardise the offer of fertility preservation techniques to all patients with an infertility risk > 50% (Fig. 2). In any case, it should be noted that the risk of gonadal insufficiency for each specific case can be difficult to calculate, and some researchers propose a somewhat lower limit when considering the use of such techniques (> 30%) [41], especially for patients in the older age groups. Additionally, when using the proposed classifications, differences according to patient sex should be considered, and the classifications should be updated when there are substantial changes in the regimens used.

In very high-risk patients, it is important to consider the possibility of using the experimental options described (IVM of primordial follicles) in the medium- to long-term, as in these cases, there is enough time for tissues to fully develop, thus potentially avoiding the need for ovarian tissue reimplantation. Fertility preservation techniques, particularly oocyte vitrification, may be considered during follow-up for young women who have completed their treatment and in whom premature depletion is expected, even if they still have an ovarian reserve.

In general, fertility preservation techniques are first-line treatments, although they can also be applied to young patients in relapse if they have maintained a good gonadal reserve and have good curative expectations.

### Adult patients with cancer

#### Solid tumours

The treatment of non-metastatic colon cancer requires surgery and adjuvant chemotherapy at stage III. The treatment of rectal cancer also includes radiotherapy as an adjuvant or neoadjuvant treatment with chemotherapy. In principle, colon cancer surgery has no impact on fertility; however, pelvic surgery for rectal cancer can affect women’s fertility [42]. Chemotherapy with 5-fluorouracil or capecitabine has no severe effect on fertility; however, oxaliplatin has a moderate effect. In women, the technique of choice for preserving fertility is oocyte cryopreservation. If the need for treatment does not allow time for this technique, ovarian cortex cryopreservation is indicated. If only pelvic radiotherapy is performed, ovarian transposition may be considered, although an alternative preservation technique is recommended as this technique is not always safe. In men, the indicated fertility preservation method is semen cryopreservation.

Ablative therapy with I^131^ may be necessary for the treatment of thyroid cancer. I^131^ produces oligomenorrhea in > 20% of women but has no long-term effects; therefore, it is not necessary to recommend any fertility preservation technique. In men, the administration of a single ablative dose of I^131^ (3 GBq, equivalent to an absorbed testicular dose < 0.1 Gy) has transient effects on spermatogenesis or male fertility. However, gonadal damage may be cumulative when multiple administrations are required, and therefore cryopreservation is advised in male patients with metastases or pelvic involvement, who can receive up to 1 Gy [43, 44].

Surgery is the primary treatment for sarcomas. Radiotherapy may be indicated for high-grade or locally advanced tumours. Because the most common locations are the limbs, pelvis and retroperitoneum, the reproductive organs are included or close to the irradiation field in many cases. Chemotherapy with alkylating agent regimens is the main treatment for many sarcomas [45]. Therefore, in women, oocyte cryopreservation is recommended if time allows, and, if not, ovarian cortex cryopreservation may be considered along with ovariopexy, when possible. In men, the best option is semen cryopreservation.

The initial treatment for testicular cancer is orchiectomy. Adjuvant treatment with platinum-based chemotherapy or radiotherapy reduces the risk of relapse and is recommended based on risk assessment. Currently, chemotherapy is the treatment of choice, and semen cryopreservation is recommended before its initiation, either before or after orchiectomy. Semen quality decreases due to testicular cancer itself, and unilateral orchiectomy can further reduce sperm count.

Breast cancer is the most common neoplasm during reproductive age [46]. The choice of neo- or adjuvant treatment depends on the tumour subtype and the risk of relapse. Chemotherapy is the treatment with the greatest effect on fertility. The fertility effects of different chemotherapy protocols is variable, as shown in Fig. 1. Oocyte vitrification is the technique of choice to preserve fertility if there is time for ovarian stimulation. No increased risk of relapse has been observed after ovarian stimulation with gonadotropins, simultaneously with letrozole or tamoxifen, although there are few published reports [47]. Ovarian cortex cryopreservation is indicated in cases in which there is no time for stimulation. When hereditary cancer is suspected, the use of this technique is more controversial; it should be performed when no other technique is possible, and graft excision should occur once pregnancy is achieved and before the age of 40. When previously described options cannot be undertaken, other options of an experimental nature for which results are available include the use of GnRH agonists or in vitro oocyte maturation [24].

As previously mentioned, adjuvant hormone therapy in breast cancer does not compromise fertility, but the duration of this therapy means that some patients have no chance of pregnancy after its completion. The results of the POSITIVE study, which examines whether temporarily interrupting adjuvant endocrine therapy to allow for pregnancy increases the risk of relapse in young patients with luminal breast cancer, will elucidate whether this strategy is possible [48].

In epithelial ovarian cancer, surgery involves hysterectomy and bilateral salpingo-oophorectomy. Fertility-sparing surgery (unilateral oophorectomy with correct staging) can be considered in cases of (i) stage IA tumours with low-grade histology (G1–G2) and (ii) borderline tumours [49], and it can be individually considered in (iii) patients with stage 1C1 tumours with intraoperative rupture of the tumour capsule and negative peritoneal cytology [50]. Subsequently, a thorough follow-up should be performed and, after the patient’s reproductive goals have been achieved, salpingo-oophorectomy should be performed. In stages III and IV, conservative surgery is contraindicated [51]. Oocyte vitrification is indicated in stage IA and borderline tumours due to the possibility of recurrence or bilaterality and the need to remove the contralateral ovary. Other indications are a diagnosis of low ovarian reserve, patient age, or cases in which adjuvant chemotherapy is proposed [51]. Ovarian cortex cryopreservation is not recommended because of the potential risk of tumour cell reimplantation.

In malignant ovarian germ cell tumours, unilateral salpingo-oophorectomy is the treatment of choice for young patients with early-stage tumours and may be considered in selected cases of advanced disease. Oocyte cryopreservation is indicated for patients who will be receiving chemotherapy.

In cases of cervical cancer, conservative surgical treatment (conisation and radical trachelectomy) can be considered for [46, 52] (i) stage IA1 microinvasive carcinoma without lymphovascular involvement and (ii) clinical stage IA1 epidermoid tumours and adenocarcinomas with lymphovascular invasion up to IB1 (< 2 cm), once high-grade histology has been ruled out; (iii) in some cases with stage IB2 (> 2 cm), neoadjuvant chemotherapy to reduce tumour size followed by radical trachelectomy and pelvic lymphadenectomy may be considered, although this is not a standard procedure, and long-term safety studies are lacking [50]. Oocyte vitrification is indicated for patients who will receive chemotherapy.

Endometrial cancer is treated with surgery (hysterectomy and bilateral adnexectomy). In patients with endometrioid carcinomas, stage IA G1 tumours without myometrial invasion, and if pregnancy is desired, high doses of oral medroxyprogesterone (400–600 mg/day) or megestrol acetate (160 mg) and a levonorgestrel IUD may be proposed to preserve fertility, with close follow-up [53] and surgery after the patient’s reproductive desire is fulfilled. Assisted reproduction techniques can facilitate and shorten the time required to achieve pregnancy, given the high incidence of obese and anovulatory patients. Letrozole may be added during the stimulation treatment.

#### Haematological tumours

Patients with haematological diseases require a specific approach to fertility preservation since (i) the incidences of acute lymphoblastic leukaemia (ALL) and Hodgkin’s lymphoma (HL) are very high in adolescents and young adults; (ii) many patients present with medical complications that prevent the use of standard fertility preservation techniques and, in some cases, contraindicate them; (iii) many patients will undergo haematopoietic stem cell transplantation (HSCT), which is associated with a high risk of infertility.

Among cancer therapies, alkylating agents, platinum and radiotherapy carry an increased risk of infertility. In women, infertility risk is correlated with age at the time of treatment.

In Hodgkin’s lymphoma, the two most commonly used first-line treatment regimens are the combination of Adriamycin, bleomycin, vinblastine and dacarbazine (ABVD) and, to a lesser extent, the escalated combination of bleomycin, etoposide, Adriamycin, cyclophosphamide, vincristine (Oncovin^®^), procarbazine and prednisone (BEACOPP).

The ABVD regimen rarely produces permanent sterility [54]. A small study in women treated with ABVD and radiotherapy showed no ovarian failure in women younger than 25 years and transient amenorrhea in 33% of women under 45 years of age [55]. Male patients treated with ABVD who develop oligospermia recover spermatogenesis completely after 18 months [56].

In acute leukaemia, most polychemotherapy regimens produce low gonadotoxicity. In acute myeloid leukaemia (AML), the risk of infertility is lower than in ALL. Infertility will depend on whether patients receive HSCT; therefore, fertility preservation should be considered in patients who are candidates for HSCT [57].

In terms of chronic myeloid leukaemia (CML), several retrospective studies show that in male patients treated with imatinib, there is no risk of infertility or teratogenicity. However, in women, there is an absolute indication to discontinue treatment in case of pregnancy, since clinical trials have shown the drug is teratogenic due to its off-target effect [58]. Therefore, in women who desire pregnancy, a deep and sustained major molecular response (MMR) must be achieved for 18–24 months to allow the discontinuation of TKIs, which should take place three months before conception and throughout pregnancy.

When HSCT is performed, the risk of infertility varies depending on the underlying disease, the treatment type and dose received before HSCT, the conditioning treatment administered and the patient’s age at the time of HSCT. Most conditioning treatments based on chemotherapy and radiotherapy cause infertility. The development of graft-versus-host disease can also lead to infertility.

## Subsequent follow-up and pregnancy

Currently, there are no robust data associating cancer diagnosis and treatment with an increase in complications during pregnancy. In female cancer survivors, no significant increase in miscarriage or congenital or chromosomal abnormalities has been demonstrated [59–61]. However, in a subgroup of patients who have received holocranial and especially pelvic radiotherapy, an increased likelihood of miscarriage [7, 14], second-trimester pregnancy loss, preterm delivery and low birth weight has been observed [59–63]. Among patients diagnosed with and treated for cancer during childhood, the impact of treatment on pregnancy complications such as increased metabolic risk, gestational diabetes, and hypertension remains controversial and there are conflicting results [63–65]. Regarding the association between the administration of anthracyclines and the development of cardiotoxicity, only patients with altered cardiac function showed a non-significant postpartum worsening on echocardiography and worse perinatal outcomes, including more admissions to the intensive care unit (ICU), longer hospital stays, and higher rates of labour induction, and it may be advisable to monitor these patients before pregnancy or in the first trimester [66]. When the study population comprised women, who were diagnosed and treated in early adulthood, higher rates of late miscarriage, caesarean section, preterm delivery, low birth weight, neonatal distress and admission to the neonatal ICU were observed. However, no increase in congenital anomalies, perinatal death, antepartum haemorrhage, premature rupture of membranes or failure of labour to progress was observed [67].

The effect of pregnancy on cancer is particularly complex in cases of breast cancer, especially in patients with hormone receptor-positive tumours. The current belief is that there is no association between pregnancy and the risk of recurrence or increased mortality in these patients, regardless of their hormone receptor status [68]. In 2015, Goldrat et al. published a retrospective multicentre study in which they observed no differences in disease prognosis between patients who conceived spontaneously and those who did so through assisted reproduction techniques [69].

In men undergoing semen cryopreservation, it is advisable to use contraceptive measures from the beginning of cancer treatment until 18–24 months after its completion. Clinical and analytical follow-up (semen analysis, FSH, LH, testosterone) is recommended until the resolution of the underlying disease; additional recommendations include informing the attending physicians of the patient's underlying disease and making an epicrisis report on residual fertility 3–5 years after the end of treatment. The degree of fertility recovery will determine whether the semen should be kept or cryopreservation can be ended. If there is a reproductive desire and the semen quality has returned to normal, natural reproduction can be allowed. If the patient has become azoospermic or severely oligozoospermic, the use of cryopreserved semen with assisted reproduction techniques appropriate for the quality of the semen can be considered. The decision to use cryopreserved gametes or those produced after successful treatment should be individualised according to the initial quality of the gametes and the clinical circumstances at the time of freezing compared to the current situation and parameters. The analysis of sperm DNA fragmentation and/or aneuploidy can contribute to the most favourable decision.

## Regulatory issues

### Specific criteria for access to fertility preservation techniques

The current legal framework is set out in Royal Decree 1030/2006. In the field of gamete or pre-embryo preservation for deferred autologous use according to medical indications with the aim of preserving fertility in situations associated with particular disease processes, the general criteria for access to assisted human reproduction (AHR) treatments must be met, except for factors addressed in the specific criteria for this technique, which will prevail over general criteria.

The common portfolio of AHR services (Order SSI/2065/2014, of 31 October) states that fertility preservation techniques will be performed in patients with a possible risk of loss of their reproductive capacity associated with exposure to gametotoxic treatments or pathological processes with a proven risk of premature ovarian failure or primary testicular failure. The transfer of cryopreserved gametes or pre-embryos will be performed in women under 50 years of age as long as they do not have any condition for which pregnancy could entail a serious and uncontrollable risk to both their health and that of their possible offspring.

At present, techniques are to be performed exclusively based on the medical indications in the public service portfolio and are not to be provided only in cases of the patient's request for deferred use.

Gametes may be cryopreserved in authorised gamete banks for at least the duration of the donor's lifetime, so a priori*,* there is no time limit for their conservation. The samples will remain at the disposal of the sperm and oocyte bank if the donors cannot be contacted after two years have elapsed since the samples were deposited.

### Information and consent

The freezing of semen and oocytes entails obligations and conditions of which patients must be informed, and specific consent must be obtained from patients with a medical indication who express interest in fertility preservation. In minors, the consent of the child and his or her parents or legal guardians is required.

A signed request is required to remove cryopreserved samples and to destroy them. Although such discussions may generate some apprehension on the part of the doctor, it is necessary to inform patients of the legal conditions regulating the use of their gametes in the event of death. In such cases, for semen to be used to fertilise the patient’s wife or partner, there must be prior consent in a public deed or will, and the semen must be used within twelve months of the patient’s death. In the case of a married man, the birth of a child conceived in the indicated manner shall produce the legal effects derived from the marital affiliation. In the case of an unmarried man, such consent shall serve as the basis to initiate proceedings under article 49 of the Civil Registry Law (for the registration of natural filiation) without prejudice of legal action to establish paternity. Consent is presumed to have been given when an assisted reproduction process is initiated prior to the death of a male partner.

## Conclusions

With the improvements in diagnosis, treatment and survival rates in patients with cancer that have been attained in recent years, quality of life is becoming increasingly important. Reduction or loss of fertility as a result of cancer or cancer treatment impairs the quality of life of survivors. The development of various fertility preservation techniques has made it possible to offer reproductive counselling to these patients. This counselling must be carried out in a multidisciplinary way in specialised centres. The degree of gonadotoxicity and, in the case of women, the patient’s age and state of her ovarian reserve, are decisive factors for the indication of a specific fertility preservation procedure. Moreover, it should always be considered that in patients with cancer, the fertility preservation procedure is subject to assessments of the patient's prognosis and functional status.

Oocyte vitrification is currently the technique of choice in most cases. However, the most appropriate technique depends on the patient's characteristics, disease and treatment, and an individualised approach is mandatory. In adult and young males with advanced pubertal development, the usual technique is semen cryopreservation. The continuous development of different techniques results in the establishment of new experimental procedures. It is thus necessary to create international registries of the outcomes and follow-up data for each technique.
